# Screening of Olive Biodiversity Defines Genotypes Potentially Resistant to *Xylella fastidiosa*

**DOI:** 10.3389/fpls.2021.723879

**Published:** 2021-08-16

**Authors:** Stefano Pavan, Marzia Vergine, Francesca Nicolì, Erika Sabella, Alessio Aprile, Carmine Negro, Valentina Fanelli, Michele Antonio Savoia, Vito Montilon, Leonardo Susca, Chiara Delvento, Concetta Lotti, Franco Nigro, Cinzia Montemurro, Luigi Ricciardi, Luigi De Bellis, Andrea Luvisi

**Affiliations:** ^1^Department of Soil, Plant and Food Science, University of Bari “Aldo Moro”, Bari, Italy; ^2^Department of Biological and Environmental Sciences and Technologies, University of Salento, Lecce, Italy; ^3^Department of Agriculture, Food, Natural Resources and Engineering, University of Foggia, Foggia, Italy

**Keywords:** olive, *Xylella fastidiosa*, genetic diversity, DNA markers, resistance

## Abstract

The recent outbreak of the Olive Quick Decline Syndrome (OQDS), caused by *Xylella fastidiosa* subsp. *pauca* (*Xf*), is dramatically altering ecosystem services in the peninsula of Salento (Apulia Region, southeastern Italy). Here we report the accomplishment of several exploratory missions in the Salento area, resulting in the identification of thirty paucisymptomatic or asymptomatic plants in olive orchards severely affected by the OQDS. The genetic profiles of such putatively resistant plants (PRPs), assessed by a selection of ten simple sequence repeat (SSR) markers, were compared with those of 141 Mediterranean cultivars. Most (23) PRPs formed a genetic cluster (K1) with 22 Italian cultivars, including ‘Leccino’ and ‘FS17’, previously reported as resistant to *Xf*. The remaining PRPs displayed relatedness with genetically differentiated germplasm, including a cluster of Tunisian cultivars. Markedly lower colonization levels were observed in PRPs of the cluster K1 with respect to control plants. Field evaluation of four cultivars related to PRPs allowed the definition of partial resistance in the genotypes ‘Frantoio’ and ‘Nocellara Messinese’. Some of the PRPs identified in this study might be exploited in cultivation, or as parental clones of breeding programs. In addition, our results indicate the possibility to characterize resistance to *Xf* in cultivars genetically related to PRPs.

## Introduction

*Xylella fastidiosa* (*Xf*) is a Gram-negative bacterium affecting more than 350 plant species, including tree crops of major economic importance (Zarco-Tejada et al., 2018). Disease symptoms include leaf scorching, leaf and twig wilting and, in a second phase, tree die-back, due to the formation of bacterial biofilms in xylematic vessels (Purcell and Hopkins, 1996). In the peninsula of Salento (Apulia Region, southeastern Italy), *Xf* subsp. *pauca* is responsible of the outbreak of the Olive Quick Decline Syndrome (OQDS), resulting in a massive socio-economic impact in the last 10 years (Loconsole et al., 2014; Saponari et al., 2019). Recent data indicate the occurrence of about 5 million unproductive, heavily infected or dead, olive trees in Salento, leading to a loss of about 10% of Italian olive oil (White et al., 2020). According to spatially explicit economic models, *Xf* may impact Italian olive growers from 1.5 to 5.9 billion euros in the next 50 years and cause further economic losses by affecting cultural heritage and landscape values (Schneider et al., 2020).

*Xf* can be limited by agronomic practices affecting the vector *Philaenus spumarius* (Liccardo et al., 2020; Schneider et al., 2020) and, possibly, by acting on the soil microbiome (Fernández-González et al., 2019). However, replanting with resistant genotypes appears as the most feasible and promising strategy to control the bacterium. Here, we use the term ‘resistance’ in a broad sense, including both resistance *sensu stricto*, i.e., the ability to limit pathogen proliferation, and tolerance, i.e., the capacity to limit the development of symptoms regardless of the level of pathogen colonization (Pagán and García-Arenal, 2018). So far, the cultivars ‘Leccino’ and ‘FS17’ (also referred to as ‘Favolosa’) were shown to display resistance to *Xf* (Baù et al., 2017; Boscia et al., 2017). Therefore, the current regulatory measures only allow ‘Leccino’ and ‘FS17’ for olive reconversion in infected areas. It was shown that resistance in ‘Leccino’ and ‘FS17’ is associated with reduced levels of bacterial growth in xylematic vessels (Saponari et al., 2016; Giampetruzzi et al., 2020).

Screening olive biodiversity for response to *Xf* may lead to the identification of resistant genotypes in addition to ‘Leccino’ and ‘FS17’. In turn, this would represent the basis for the reconversion of infected areas into more complex, resilient, agro-ecosystems, and the selection of cultivars merging resistance with other economically important traits, such as agronomic performance and oil quality. So far, screenings were limited to a few Italian cultivars (Baù et al., 2017; Boscia et al., 2017). None of them displayed resistance levels comparable to those of ‘Leccino’ and ‘FS17’; however, a wide range of variation was detected. Intermediate resistance was reported for ‘Frantoio’, ‘Toscanina’, ‘Termite di Bitetto’, ‘Maiatica’, ‘Dolce di Cassano’, ‘Oliastro’, ‘Nociara’, and ‘Nocellara Etnea’ (Baù et al., 2017; Boscia et al., 2017).

In Salento, about 85% of the olive trees refer to two cultivars, ‘Cellina di Nardò’ and ‘Ogliarola Salentina’, both extremely susceptible to *Xf* (Luvisi et al., 2017). In heavily infected orchards, this creates a spooky scenario of desiccated trees, in which putatively resistant genotypes, asymptomatic or paucisymptomatic, stand out. Here, we present the results of a study mainly aimed to: (i) identify genotypes putatively resistant to *Xf*, through exploratory missions conducted in Salento; (ii) establish genetic relationships between putatively resistant genotypes and a large panel of cultivars occurring in the Mediterranean region.

## Materials and Methods

### Exploratory Missions and Characterization of Putatively Resistant Plants

Exploratory missions were carried out in the Autumns of 2019 and 2020 upon report, by local growers, of asymptomatic or paucisymptomatic plants (reported plants or RPs) in olive orchards heavily affected by *Xf*. RPs were evaluated for disease severity (S) using the pathometric scale previously described by Luvisi et al. (2017), ranging from 0 (no symptoms) to 3 (canopy with uniformly distributed desiccated branches). Three control plants (CPs) randomly chosen in the proximity of each RP were also evaluated for disease severity. A RP was deemed as a putatively resistant plant (PRP) if S_CP_ − S_RP_ (ΔS) ≥ 1.5, with S_CP_ being the average value of S calculated from its three CPs.

For bacterial quantification, 1-year-old branches (8–10), with attached leaves, were collected from the apical, median, and basal portions of the canopy of PRPs and CPs. Branches from each triad of CPs were pooled. Approximately one gram of leaf petioles was transferred into an extraction bag (BIOREBA, Switzerland) and processed as described by Sabella et al. (2018). DNA was extracted according to Edwards et al. (1991) and used as template for *Xf* detection by the TaqMan real-time PCR protocol with the XF-F/R primers and the XF-P probe (Harper et al., 2010). Each reaction consisted of 5 μL from a 20 ng μL^−1^ dilution of DNA, 12.5 μL of TaqMan Environmental Master Mix 2.0 (Applied Biosystems, CA, United States), 0.4 μM of each primer, 0.2 μM of TaqMan probe, and ultrapure DNase/RNase-free water (Carlo Erba Reagents, Italy) in a total volume of 25 μL. The cycling conditions were those described by Harper et al. (2010). *Xf* concentration (C), expressed as bacterial cfu ml^–1^, was inferred from Cq values using a standard curve with dilutions ranging from 10^2^ to 10^7^ cfu ml^–1^, as described by D’Attoma et al. (2019). The minimum value of 10^2^ cfu ml^–1^ was assigned to negative samples as detection limit, whereas the maximum value of 10^7^ cfu ml^–1^ was assigned to samples in which the bacterium could not be detected due to extremely advanced plant desiccation. The parameter ΔC, given by C_CP_ – C_PRP_, was finally calculated for each PRP.

Disease symptoms and bacterial concentrations recorded for PRPs falling in the genetic cluster K1, which was identified by hierarchical clustering, were compared with those of respective controls, using the Wilcoxon signed-rank test.

### SSR Marker Analysis and Assessment of Genetic Relationships With Known Cultivars

DNA of PRPs was used as template to perform Simple Sequence Repeat (SSR) marker analysis, using a set of 10 primer combinations previously reported to be effective in differentiating olive genetic resources (Supplementary Table 1; Sefc et al., 2000; Carriero et al., 2002; De La Rosa et al., 2002; Pasqualone et al., 2016). PCR reactions were performed following the protocol described by Spadoni et al. (2019). Amplicons were resolved with an internal size standard (GeneScan 500 LIZ; Applied Biosystems) by capillary electrophoresis on an ABI 3130 Genetic Analyzer (Applied Biosystems, CA, United States). SSR allele sizes were estimated using the software GeneMapper v.3.7. The informativeness of each SSR marker was estimated through the polymorphic information content (PIC) index, which was calculated using the software Cervus v.3.0.7 (Marshall et al., 1998).

Marker profiles were merged with those of Mediterranean cultivars, available from previous studies (Alba et al., 2009; Boucheffa et al., 2017; di Rienzo et al., 2018; Miazzi et al., 2020; Debbabi et al., 2021). UPGMA hierarchical clustering was performed using the Nei’s distance and the ape R package (Paradis et al., 2004). Parametric clustering was performed using the software STRUCTURE v.2.3.4. (Pritchard et al., 2000) for a number of sub-populations (K) ranging from 1 to 10, using 10 runs for each K, a burn-in period of 50,000, and 100,000 Markov chain Monte Carlo iterations. The software Structure Harvester (Earl and vonHoldt, 2012) was used to infer the value of K best describing genetic structure, based on the ΔK statistics (Evanno et al., 2005).

### Characterization of Cultivars for Response to *Xf*

Four cultivars displaying genetic similarity with PRPs (‘Nocellara Messinese’, ‘Frantoio’, ‘Bella di Spagna’, ‘Pendolino’) were represented at the experimental orchard ‘Murrone’, located in Caprarica di Lecce (Province of Lecce, Italy, 40°15′59″N 18°14′52″E), together with the susceptible controls ‘Cellina di Nardò’ and ‘Ogliarola Salentina’, and the resistant control ‘Leccino’. The orchard, planted in 2015, is heavily affected by *Xf*. Disease severity and bacterial quantification were assessed in Autumn 2020 on eight biological replicates for each cultivar, following the methodologies described above. Significant differences among cultivars were assessed using the Wilcoxon rank-sum test and the false discovery rate (FDR) correction to control for Type I error.

## Results

### Exploratory Missions and Characterization of Putatively Resistant Plants

**FIGURE 1:**
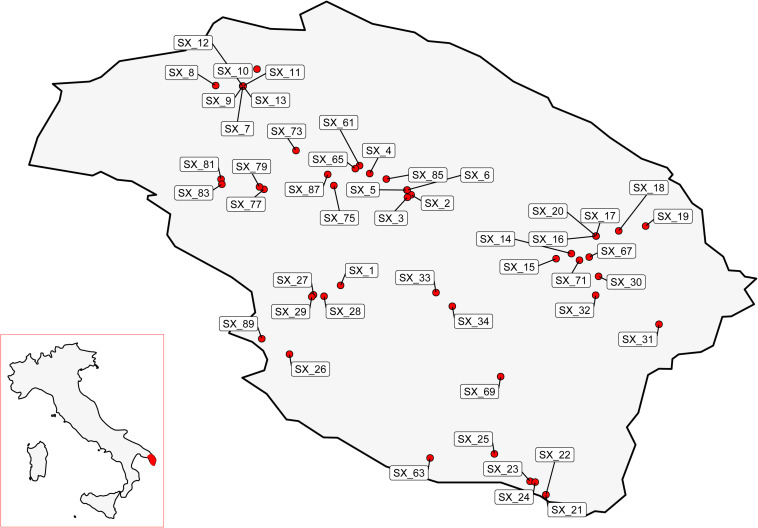
Localization, in the province of Lecce, Salento, of the plants screened in this study.

**FIGURE 2:**
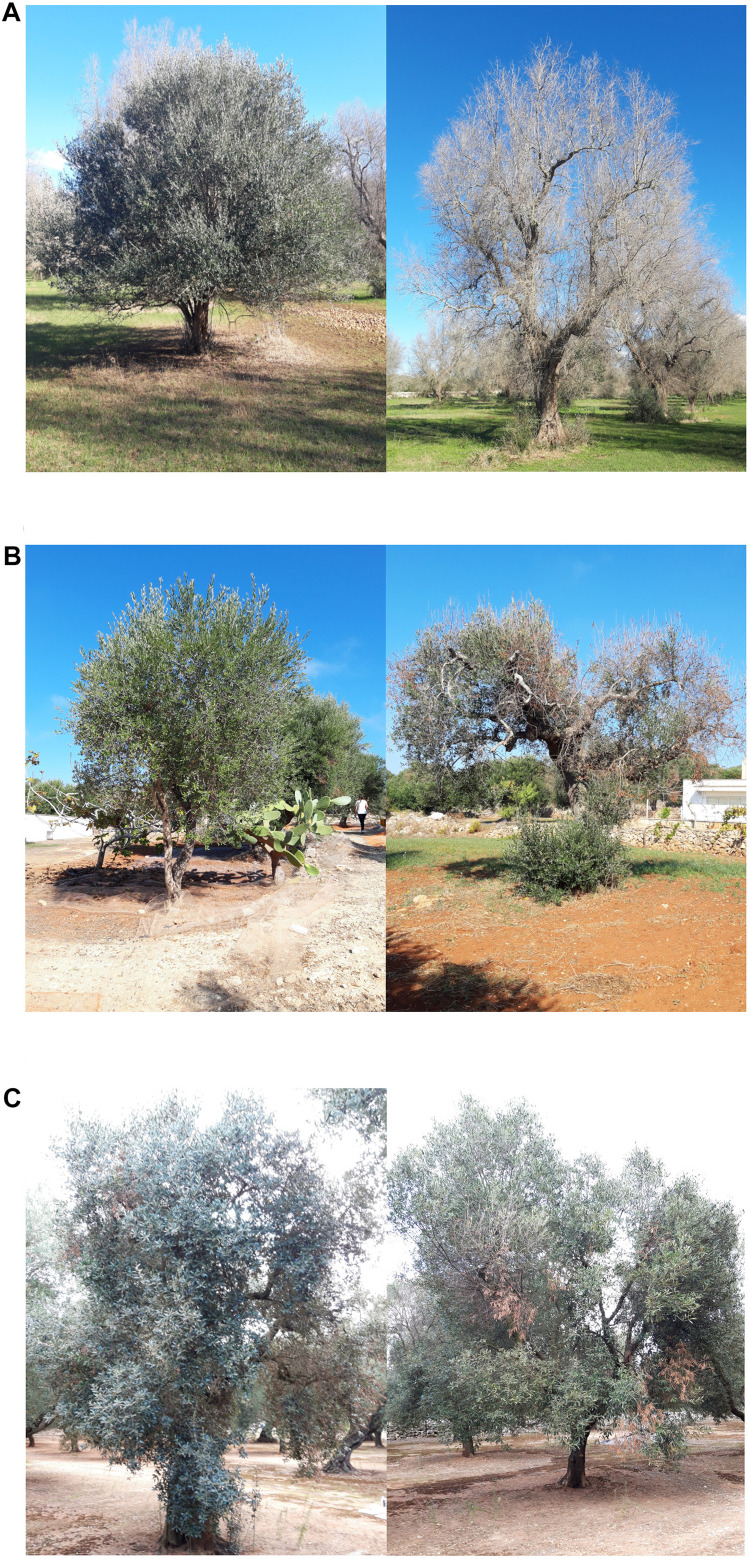
Phenotypes of plants monitored in this study (left) and respective controls (right) for **(A)** ΔS = 3, **(B)** ΔS = 2, and **(C)** ΔS = 1.

Exploratory missions were carried out in the Province of Lecce (Salento) for a periodical screening of 49 plants reported by local farmers to stand out in orchards heavily infected by *Xf* (Figure 1). For each plant, difference in disease severity with neighboring control plants (ΔS) was assessed (Figure 2). Thirty putative resistant plants (PRPs) were selected, which were associated, compared to control plants (CPs), with a difference in disease severity (ΔS) ≥ 1.5. Twelve of them also appeared asymptomatic (*S* = 0) (Table 1). The distribution of S and C values associated with PRPs and CPs are reported in Supplementary Figure 1.

**TABLE 1:** Features of putatively resistant plants (PRPs) identified in this study.

**PRP code**	**Locality**	**Approximate age (years)**	***Xf* concentration (C) in PRPs (cfu/ml)**	***Xf* concentration (C) in CPs (cfu/ml)**	Δ**C**	**Syndrome severity (S) in PRPs**	**Syndrome severity (S) in CPs**	Δ**S**
SX_32	Muro Leccese	70	7.91E + 05	8.63E + 05	7.20E + 04	0.00	3.00	3.00
SX_26	Gallipoli	30	3.51E + 03	1.09E + 05	1.05E + 05	0.13	2.75	2.63
SX_1	Seclì	70	8.78E + 03	6.40E + 04	5.52E + 04	0.00	2.50	2.50
SX_5	Lequile	40	5.15E + 03	5.10E + 04	4.59E + 04	0.00	2.50	2.50
SX_34	Cutrofiano	50	1.71E + 05	1.00E + 07	9.83E + 06	0.00	2.50	2.50
SX_65	San Pietro in Lama	25	2.10E + 03	5.28E + 05	5.26E + 05	0.25	2.75	2.50
SX_71	Bagnolo del Salento	40	7.41E + 04	1.00E + 07	9.93E + 06	0.25	2.75	2.50
SX_77	Copertino	30	1.52E + 03	1.39E + 05	1.37E + 05	0.25	2.75	2.50
SX_25	Presicce	100	1.00E + 02	2.37E + 04	2.36E + 04	0.75	3.00	2.25
SX_28	Sannicola	20	3.54E + 04	6.59E + 05	6.24E + 05	0.25	2.50	2.25
SX_75	Copertino	25	1.00E + 02	1.00E + 07	1.00E + 07	0.50	2.75	2.25
SX_79	Copertino	25	3.31E + 04	1.03E + 05	6.99E + 04	0.25	2.50	2.25
SX_81	Leverano	50	1.00E + 02	1.25E + 03	1.15E + 03	0.75	3.00	2.25
SX_83	Leverano	25	7.70E + 03	2.20E + 04	1.43E + 04	0.50	2.75	2.25
SX_89	Gallipoli	200	1.00E + 02	6.99E + 05	6.99E + 05	0.25	2.50	2.25
SX_61	San Pietro in Lama	25	1.00E + 02	1.22E + 06	1.22E + 06	0.13	2.25	2.13
SX_2	Lequile	30	4.19E + 05	1.00E + 07	9.58E + 06	0.00	2.00	2.00
SX_3	Lequile	30	2.80E + 03	7.32E + 03	4.52E + 03	0.00	2.00	2.00
SX_4	Lequile	50	2.87E + 04	9.64E + 04	6.77E + 04	0.00	2.00	2.00
SX_6	Lequile	40	6.03E + 03	5.10E + 04	4.50E + 04	0.50	2.50	2.00
SX_24	Morciano di Leuca	50	4.00E + 03	3.83E + 05	3.79E + 05	0.50	2.50	2.00
SX_27	Sannicola	100	1.00E + 02	8.75E + 05	8.75E + 05	1.00	3.00	2.00
SX_87	Copertino	30	2.36E + 04	3.10E + 06	3.08E + 06	0.75	2.75	2.00
SX_11	Campi Salentina	50	3.66E + 03	3.77E + 04	3.40E + 04	0.00	1.75	1.75
SX_8	Guagnano	40	2.73E + 05	1.00E + 07	9.73E + 06	0.00	1.50	1.50
SX_12	Campi Salentina	40	8.91E + 04	3.77E + 04	5.14E + 04	0.00	1.50	1.50
SX_29	Sannicola	50	4.41E + 03	8.55E + 04	8.11E + 04	1.25	2.75	1.50
SX_31	Minervino	80	3.13E + 05	1.00E + 07	9.69E + 06	0.75	2.25	1.50
SX_33	Cutrofiano	10	1.04E + 03	6.43E + 05	6.42E + 05	0.00	1.50	1.50
SX_67	Bagnolo del Salento	25	1.00E + 02	7.30E + 05	7.30E + 05	0.00	1.50	1.50

*For each PRP, information is provided on the locality in which it was identified, approximate age, Xf concentration (C), and syndrome severity (S). In addition, C and S values displayed by control plants (CPs) associated with each PRP are also reported, together with the values of ΔC (given by C_*CP*_ − C_*PRP*_) and ΔS (given by S_*CP*_ − S_*PRP*_). The table is sorted according to decreasing ΔS values.*

### PRP Fingerprinting and Genetic Relationships With Known Cultivars

The thirty identified PRPs were genotyped with ten short sequence repeat (SSR) markers previously reported as highly polymorphic in olive germplasm (Sefc et al., 2000; Carriero et al., 2002; De La Rosa et al., 2002; Pasqualone et al., 2016). Identical DNA fingerprints were found for the two PRPs SX_77 and SX_79, detected in the same locality (Copertino), and for the three PRPs SX_65, SX_81, and SX_89), detected in different localities (San Pietro in Lama, Leverano, and Gallipoli) (Table 1).

SSR fingerprints obtained for the PRPs were compared with those of 141 cultivars from several Mediterranean Countries, taking profit of SSR profiles available from previous studies (Alba et al., 2009; Boucheffa et al., 2017; di Rienzo et al., 2018; Miazzi et al., 2020; Debbabi et al., 2021). A total of 124 alleles was detected (Supplementary Table 2) with an average of 12.4 alleles per locus. PIC index values ranged from 0.49 to 0.90 with an average of 0.73, indicating high informativeness on DNA polymorphism of the selected SSR marker loci (Supplementary Table 2). None of the PRPs displayed a marker profile identical to the resistant cultivars ‘Leccino’ and ‘FS17’, or to the susceptible cultivars ‘Cellina di Nardò’ and ‘Ogliarola Salentina’.

**FIGURE 3:**
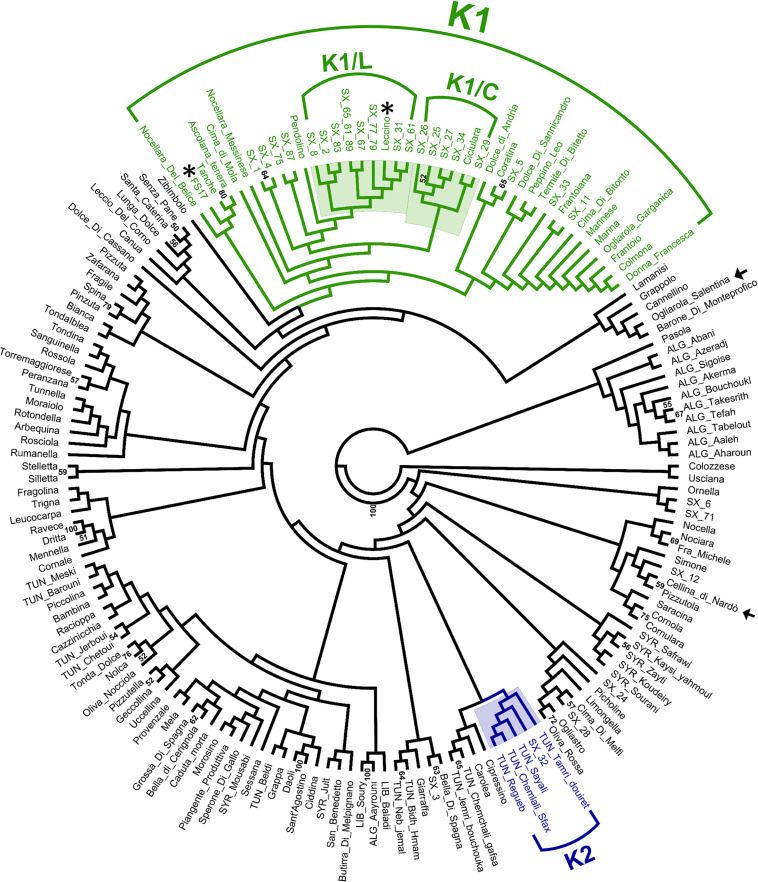
UPGMA clustering based on short sequence repeat (SSR) DNA fingerprints of 30 putatively resistant plants and 141 olive cultivars. The cluster K1 (with the sub-clusters K1/L and K1/C) and the cluster K2 are highlighted. Bootstrap values > 50 are shown. The resistant and susceptible control cultivars are indicated with asterisks and arrows, respectively.

Hierarchical clustering (Figure 3) revealed the occurrence of a genetic cluster (K1) grouping most (23) PRPs with 22 Italian cultivars, including the resistant cultivars ‘Leccino’ and ‘FS17’. The PRPs SX_2, SX_8, SX_31, SX_61, SX_67, and SX_83, together with the two groups of putatively identical PRPs SX_77_79 and SX_65_81_89, formed a sub-cluster (K1/L) of genotypes closely related with ‘Leccino’, thus suggesting the occurrence of clonal variation around this cultivar. Five PRPs (SX_25, SX_26, SX_27, SX_29, and SX_34) formed another sub-cluster (K1/C) of genotypes closely related to the cultivar ‘Ciciulara’. PRPs not included in K1 were scattered throughout the dendrogram. Notably, the PRP SX_32, associated with the highest ΔS (3), formed a cluster (K2) with several Tunisian cultivars (‘Regueb’, ‘Chemlali Sfax’, ‘Sayali’, and ‘Tamri Douiret’), thus suggesting that this plant might have an allochtonous origin.

Parametric analysis indicated a model with three sub-populations (Subpop_1–3) as the most appropriate to describe genetic structure (Supplementary Figure 2). In accordance with the results of hierarchical clustering, most individuals grouped in K1 and K2 were mainly referable to Subpop_1 and Subpop_2, respectively (Supplementary Table 3).

PRPs included in K1 displayed significantly lower bacterial colonization than respective controls (*p* = 2.38 ^∗^ 10^–7^) (Supplementary Figure 3). As for the remaining PRPs, they were characterized by highly variable ΔC values, ranging from –5.14^∗^10^4^ (SX_12) to 9.93^∗^10^6^ (SX_71) (Table 1).

### Response of Four Cultivars Displaying Genetic Similarity With PRPs

**FIGURE 4:**
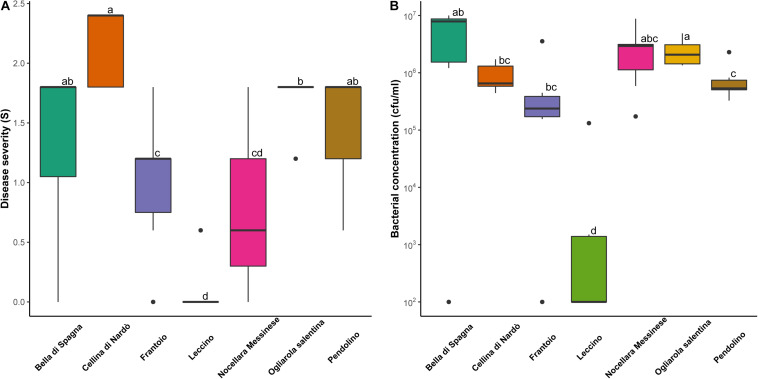
Distribution of **(A)** disease severity and **(B)** bacterial concentration observed for eight biological replicates of the following olive cultivars: ‘Bella di Spagna’, ‘Cellina di Nardo’, ‘Frantoio’, ‘Leccino’, ‘Nocellara Messinese’, ‘Ogliarola Salentina’, and ‘Pendolino’. Different letters indicate significant differences following the application of the Wilcoxon rank-sum test (*p* < 0.05).

Among the cultivars showing genetic similarity with PRPs, four were replicated in a 5-year-old experimental orchard heavily infested by *Xf*, and therefore could be evaluated for response to the bacterium: ‘Pendolino’ and ‘Frantoio’, and ‘Nocellara Messinese’, included in the cluster K1; ‘Bella di Spagna’, related to the plant SX_3. Disease severity was significantly lower in ‘Nocellara Messinese’ and ‘Frantoio’ than in the susceptible control cultivars ‘Ogliarola Salentina’ and ‘Cellina di Nardò’ (Figure 4A). Significant differences were also found between the resistant control ‘Leccino’, displaying the lowest level of symptoms, and all the other cultivars (Figure 4A). ‘Leccino’ also exhibited the lowest level of bacterial colonization (Figure 4B).

Correlation was found between the level of symptoms and bacterial concentration (*p* < 0.05) in individual trees. Consistently, the two outlier individuals within the cultivars ‘Bella di Spagna’ and ‘Frantoio’, displaying no visible symptoms, were also characterized by undetectable *Xf* colonization.

## Discussion

There is an urgent need to reconvert olive orchards exposed to *Xf* with resistant germplasm. So far, only two cultivars, ‘Leccino’ and ‘FS17’, were reported as resistant to *Xf* (Boscia et al., 2017). Although to a low extent, both of them support bacterial colonization and may display disease symptoms (Boscia et al., 2017; Giampetruzzi et al., 2020); in addition, their performance under pathogen pressure, with respect to agronomic, qualitative and technological traits, should be further investigated. In this context, the identification of further sources of resistance to *Xf* may allow the selection of cultivars increasing the complexity, and thus the resilience, of olive agro-ecosystems, and displaying superior features.

The screening of olive germplasm for response to *Xf* may be accomplished through the set-up of experimental orchards in areas heavily infected by the bacterium. This approach, besides being relatively costly, requires several months or years for the emergence of symptoms after exposure to the bacterium. In this study, we followed an alternative and rapid approach for the identification of germplasm resistant to *Xf*, based on the detection, in existing orchards, of individuals standing out among heavily symptomatic or dead trees. Some of the thirty PRPs identified in this study might deserve direct exploitation in cultivation. Therefore, we are currently proceeding with their clonal propagation and thoroughly characterization in replicated trials with respect to response to *Xf* and other main economic traits.

SSR profiles obtained for PRPs were compared with those available from previous studies, referring to a large panel of cultivars occurring in Italy and the Mediterranean area. Most (23) of the PRPs were grouped in the cluster K1, including the resistant cultivars ‘Leccino’ and ‘FS17’ (Figure 3). This indicates that the screening of cultivars in K1 might represent a valuable strategy for the identification of germplasm resistant to *Xf*.

Eight different genotypes, associated with 11 PRPs, formed the sub-cluster K1/L together with the cultivar ‘Leccino’. Noteworthy, none of these genotypes fully matched the one of ‘Leccino’, suggesting that wide clonal variation exists within germplasm identified as ‘Leccino’. Screening of such variation might lead to the selection of clones displaying higher levels of resistance and/or superior attributes with respect to other economic traits. The cultivar ‘Ciciulara’, forming another sub-cluster (K1/C) with five closely related PRPs, was previously characterized in Salento in the framework of a Regional Project aimed at the recovery of minor olive germplasm (Miazzi et al., 2020). Interestingly, ‘Ciciulara’ is clearly distinct at the morphological level from ‘Leccino’, as it displays very large drupes that do not turn completely black at maturity.

All the cultivars displaying similarity with the PRPs identified in this study represent obvious targets for future investigations aiming to the characterization of new sources of resistance to *Xf*. Here, we assessed the response of three other cultivars of this kind, namely ‘Frantoio’, ‘Nocellara Messinese’, and ‘Pendolino’, included in K1, and ‘Bella di Spagna’, related to SX_3. Both ‘Nocellara Messinese’ and ‘Frantoio’ displayed significantly lower symptoms than the susceptible controls ‘Ogliarola Salentina’ and ‘Cellina di Nardò’, although none of them displayed the same level of resistance of ‘Leccino’. This is consistent with preliminary studies indicating partial resistance in ‘Frantoio’ (Boscia et al., 2017; Luvisi et al., 2017).

Of main interest it would be the characterization of the response of Tunisian cultivars closely related to the plant SX_32, which displayed no symptoms and the highest possible ΔS value (3). In general, the exploration of olive gene pools occurring outside Italy might lead to the identification of new sources of resistance to *Xf*. In this respect, we highlight previous studies reporting regional stratification of olive biodiversity in the Mediterranean region (Belaj et al., 2012; El Bakkali et al., 2013) and the occurrence of hotspots of genetic diversity in specific areas (Hosseini-Mazinani et al., 2014; Deddabi et al., 2020).

Based on *Xf* quantification in xylematic vessels, we showed that reduced symptoms in ‘Leccino’ and PRPs included in the cluster K1 might be due to (active or passive) defense mechanisms limiting bacterial proliferation. This is consistent with preliminary studies indicating partial resistance in ‘Frantoio’ (Boscia et al., 2017). In contrast, tolerance to the bacterium might be at the basis of the response of ‘Nocellara Messinese’, which exhibits markedly lower symptoms than ‘Ogliarola Salentina’ and ‘Cellina di Nardò’, but similar levels of *Xf* colonization. Similarly, tolerance might characterize SX_32, displaying a similar level of bacterial colonization with respect to the control (Table 1).

Overall, the results of this study may guide future efforts aimed to the selection of cultivars displaying resistance to *Xf*. High-throughput genotyping by whole or partial genome sequencing (Taranto et al., 2018; Pavan et al., 2020) might allow the collection of more comprehensive fingerprint data on the clones considered in this work, and the characterization of loci associated with resistance to *Xf* by means of conventional or extreme phenotype genome-wide association studies (Yang et al., 2015).

## Data Availability Statement

The raw data supporting the conclusions of this article will be made available by the authors, upon reasonable request to the corresponding author.

## Author Contributions

SP and AL conceived the research. MV, FNic, ES, AA, and CN performed exploratory missions and identified putatively resistant plants. VM, LS and FNig contributed to the evaluation of cultivars for response to *Xylella fastidiosa*. VF, MS, and CM contributed to SSR analysis. SP and CD performed data analysis. SP wrote the first draft of the manuscript. AL, LD, CL, CM, LR, and FNig critically revised the manuscript. All authors have read and approved the final manuscript.

## Conflict of Interest

The authors declare that the research was conducted in the absence of any commercial or financial relationships that could be construed as a potential conflict of interest.

## Publisher’s Note

All claims expressed in this article are solely those of the authors and do not necessarily represent those of their affiliated organizations, or those of the publisher, the editors and the reviewers. Any product that may be evaluated in this article, or claim that may be made by its manufacturer, is not guaranteed or endorsed by the publisher.

## Supplementary Material

Supplementary Figure 1 Distribution of **(A)** disease severity scores (S) and **(B)** bacterial concentration **(C)** in thirty putatively resistant plants (PRPs) and respective control plants (CPs).

Supplementary Figure 2 Parametric study of genetic structure. **(A)** Distribution of the ΔK parameter. **(B)** Genetic structure for three subpopulations (*K* = 3). Each individual is represented by a vertical line, which is partitioned into colored segments whose length is proportional to the estimated membership fraction (qi) in each subpopulation.

Supplementary Figure 3 Bacterial colonization levels in PRPs included in K1 with respect to control plants.

Supplementary Table 1 Features of the SSR loci used in this study, including the repeat motif, forward and reverse sequences, and reference in scientific literature.

Supplementary Table 2 Summary statistics for the SSR analysis. For each SSR locus, the number of alleles, allele sizes, and polymorphic information content (PIC), are indicated.

Supplementary Table 3 Membership coefficients (q_i_) of the individuals genotyped in this study for the three subpopulations identified by STRUCTURE analysis. Individuals grouped in the clusters K1 and K2 identified by hierarchical clustering are indicated.
