# Supplementary material for: Screening of Olive Biodiversity Defines Genotypes Potentially Resistant to Xylella fastidiosa
Source: Front Plant Sci. 2021 Aug 16;12:723879. doi: 10.3389/fpls.2021.723879 (PMC8415753; doi:10.3389/fpls.2021.723879)
Supplement: Supplementary Figure 1 — Distribution of (A) disease severity scores (S) and (B) bacterial concentration (C) in thirty putatively resistant plants (PRPs) and respective control plants (CPs). [file Data_Sheet_1.PDF]

Supplementary Materials

Supplementary Table 1. Features of the SSR loci used in this study, including the repeat motif, forward and reverse sequences, and reference in scientific literature.

Locus	Repeat motif	Primer Sequence (5' to 3')		Bibliographic reference
		Forward	Reverse	
DCA03	$(GA)_{19}$	cccaagcggaggtgtatattgttac	tgcttttgtgtttgagatgttg	Sefc *et al*. (2000)
DCA05	$(GA)_{15}$	aacaaatcccatacgaactgcc	cgtgttgctgtgaagaaaatcg	Sefc et al. (2000)
DCA09	$(GA)_{23}$	aatcaaagtcttccttctcatttcg	gatccttccaaaagtataacctctc	Sefc et al. (2000)
DCA13	$(CA)_{15}$	gatcagattaatgaagatttggg	aactgaacctgtgtatcttgcatcc	Sefc et al. (2000)
DCA15	$(CA)_3G(AC)_{14}$	gatcttgtctgtatatccacac	tataccttttccatcttgacgc	Sefc et al. (2000)
DCA17	$(GT)_9(AT)_7AGATA(GA)_{38}$	gatcaaattctaccaaaaatata	taatttttggcacgtagtattgg	Sefc et al. (2000)
DCA18	$(CA)_4CT(CA)_3(GA)_{19}$	aagaaagaaaaaggcagaattaagc	gttttcgtctctctacataagtgac	Sefc et al. (2000)
GAPU71b	$GA(AG)_6(AAG)_8$	gatcaaaggaagaaggggataaa	acaacaaatccgtacgcttg	Carriero et al., (2002)
GAPU101	$(GA)_8(G)_3(AG)_3$	catgaaaggagggggacata	ggcacttgttgtgcagattg	Carriero et al., (2002)
EMOL	$(GA)_{12}$	ctttccaatatgggctctcg	atggcactttacgggaaaaa	De la Rosa et al. (2002)
EMO90	$(CA)_{10}$	catccggatttcttgctttt	agcgaatgtagctttgcatgt	De la Rosa et al. (2002)

Supplementary Table 2. Summary statistics for the SSR analysis. For each SSR locus, the number of alleles, allele sizes, and polymorphic information content (PIC), are indicated.

**Locus name**	**DCA03**	**DCA05**	**DCA09**	**DCA13**	**DCA17**	**DCA18**	**GAPU71**	**GAPU101**	**EMO90**	**EMOL**
**Number of alleles**	14	10	20	9	20	18	7	7	8	11
**Allele sizes**	229, 231, 235, 237, 239, 241, 243, 245, 247, 249, 251, 253, 255, 257	194, 198, 200, 202, 204, 206, 208, 210, 212, 214	162, 164, 166, 172, 174, 176, 178, 180, 182, 184, 186, 188, 190, 192, 194, 198, 204, 206, 208, 210	116, 118, 120, 122, 124, 130, 132, 140, 156	105, 107, 109, 111, 113, 115, 117, 129, 137, 139, 141 ,143, 151, 157, 159, 165, 175, 179, 181, 185	159, 163, 165, 167, 169, 171, 173, 175, 177, 179, 181, 183, 185, 187, 191, 197, 199, 201	118, 121, 124, 127, 130, 145, 160	182, 190, 192, 198, 200, 206, 218	184, 186, 188, 190, 192, 194, 196, 198	190, 192, 194, 196, 198, 200, 202, 204, 212, 214, 228
**PIC**	0.84	0.72	0.9	0.59	0.82	0.82	0.7	0.8	0.67	0.49

Supplementary Table 3. Membership coefficients (q_i) of the individuals genotyped in this study for the three subpopulations identified by STRUCTURE analysis. Individuals grouped in the clusters K1 and K2 identified by hierarchical clustering are indicated.

Individuals	Subpop_1	Subpop_2	Subpop_3	Group in hierarchical clustering
SX_75	0.974	0.012	0.014	K1
Pendolino	0.973	0.013	0.015	K1
SX_27	0.973	0.013	0.013	K1
SX_31	0.973	0.012	0.015	K1
SX_77_79	0.973	0.012	0.015	K1
SX_34	0.972	0.014	0.014	K1
SX_61	0.972	0.013	0.015	K1
SX_65_81_89	0.972	0.012	0.016	K1
Ciciulara	0.97	0.016	0.014	K1
Frantoio	0.969	0.014	0.017	K1
SX_83	0.969	0.014	0.017	K1
Leccino	0.967	0.016	0.016	K1
Donna Francesca	0.966	0.015	0.019	K1
SX_26	0.965	0.02	0.015	K1
SX_8	0.964	0.016	0.02	K1
SX_87	0.963	0.017	0.021	K1
SX_25	0.962	0.023	0.014	K1
SX_67	0.961	0.017	0.022	K1
Colmona	0.96	0.015	0.025	K1
SX_1	0.96	0.015	0.025	K1
SX_33	0.957	0.019	0.024	K1
Ogliarola Garganica	0.955	0.018	0.028	K1
SX_4	0.913	0.061	0.026	K1
SX_2	0.908	0.044	0.048	K1
SX_29	0.891	0.045	0.064	K1
Marina	0.889	0.016	0.095	K1
Marinese	0.863	0.05	0.087	K1
Cima Di Bitonto	0.845	0.046	0.109	K1
SX_11	0.822	0.046	0.132	K1
Frantoiana	0.801	0.026	0.172	K1
Cima di Mola	0.768	0.109	0.123	K1
Nocellara Messinese	0.766	0.126	0.108	K1
Dolce di Andria	0.646	0.116	0.238	K1
Coratina	0.601	0.2	0.199	K1
SX_5	0.579	0.174	0.247	K1
Termite Di Bitetto	0.55	0.029	0.421	K1
Nocellara Del Belice	0.528	0.307	0.165	K1
FS17	0.494	0.104	0.402	K1
Peppino Leo	0.424	0.067	0.509	K1
Tanche	0.3	0.253	0.447	K1
Ascolana tenera	0.087	0.811	0.102	K1
Dolce Di Sannicandro	0.205	0.04	0.754	K1
TUN_Sayali	0.017	0.935	0.047	K2
TUN_Chemlali Sfax	0.03	0.929	0.041	K2
SX_32	0.021	0.896	0.083	K2
TUN_Regueb	0.052	0.746	0.202	K2
TUN_Tamri douiret	0.035	0.317	0.647	K2
Dolce Di Cassano	0.875	0.02	0.104	Other
Bambina	0.857	0.064	0.079	Other
Ogliarola Salentina	0.754	0.014	0.232	Other
Piccolina	0.737	0.065	0.198	Other
Senza Pane	0.682	0.041	0.277	Other
Racioppa	0.673	0.237	0.09	Other
Leccio Del Corno	0.602	0.091	0.307	Other
Santa Caterina	0.588	0.031	0.381	Other
Silletta	0.551	0.358	0.091	Other
Fragile	0.505	0.328	0.167	Other
Cornale	0.49	0.298	0.212	Other
Lunga Dolce	0.484	0.021	0.495	Other
Cima Di Melfi	0.481	0.318	0.201	Other
SX_28	0.465	0.06	0.476	Other
SX_3	0.442	0.521	0.036	Other
Stelletta	0.396	0.499	0.104	Other
Zibimbolo	0.386	0.432	0.182	Other
Caduta morta	0.371	0.302	0.327	Other
Bella di Cerignola	0.366	0.58	0.054	Other
Cazzinicchia	0.316	0.08	0.604	Other
ALG_Abani	0.01	0.975	0.015	Other
ALG_Aharoun	0.013	0.972	0.015	Other
ALG_Tefah	0.017	0.969	0.014	Other
ALG_Takesrith	0.017	0.968	0.014	Other
ALG_Tabelout	0.014	0.968	0.018	Other
ALG_Bouchoukl	0.017	0.965	0.018	Other
ALG_Azeradj	0.014	0.964	0.021	Other
ALG_Aaleh	0.019	0.961	0.021	Other
SYR_Sourani	0.02	0.959	0.021	Other
SYR_Zayti	0.022	0.953	0.025	Other
SYR_Kaysi yahmoul	0.028	0.951	0.021	Other
ALG_Aayrouni	0.029	0.939	0.031	Other
SYR_Koudeiry	0.015	0.938	0.047	Other
TUN_Chetoui	0.019	0.934	0.047	Other
LIB_Soury	0.029	0.933	0.037	Other
ALG_Sigoise	0.013	0.919	0.068	Other
TUN_Neb jemal	0.05	0.9	0.051	Other
Ogliastro	0.07	0.898	0.032	Other
Butirra Di Melpignano	0.061	0.898	0.042	Other
Giarraffa	0.052	0.897	0.051	Other
SYR_Mousabi	0.027	0.895	0.078	Other
TUN_Bidh Hmam	0.08	0.894	0.026	Other
Sperone Di Gallo	0.039	0.882	0.079	Other
TUN_Barouni	0.076	0.86	0.064	Other
Carolea	0.02	0.853	0.127	Other
Bella Di Spagna	0.127	0.851	0.023	Other
Mela	0.054	0.851	0.096	Other
ALG_Akerma	0.119	0.824	0.057	Other
Picholine	0.067	0.821	0.111	Other
TUN_Jemri bouchouka	0.045	0.814	0.142	Other
San Benedetto	0.041	0.814	0.145	Other
LIB_Baladi	0.04	0.812	0.149	Other
SYR_Jult	0.092	0.81	0.099	Other
Colozzese	0.128	0.803	0.069	Other
Sant'Agostino	0.027	0.781	0.192	Other
Cipressino	0.024	0.757	0.218	Other
Daoli	0.031	0.755	0.214	Other
SYR_Safrawi	0.032	0.738	0.23	Other
Usciana	0.022	0.738	0.24	Other
TUN_Jerboui	0.036	0.737	0.227	Other
Sessana	0.156	0.719	0.125	Other
TUN_Meski	0.035	0.708	0.256	Other
TUN_Beldi	0.227	0.682	0.091	Other
TondaIblea	0.201	0.661	0.137	Other
Grossa Di Spagna	0.207	0.644	0.15	Other
Provenzale	0.122	0.642	0.236	Other
Zafarana	0.096	0.636	0.268	Other
Oliva Rossa	0.211	0.621	0.168	Other
TUN_Chemchali gafsa	0.025	0.564	0.411	Other
Mennella	0.242	0.515	0.243	Other
Piangente Produttiva	0.132	0.447	0.421	Other
Morosino	0.063	0.425	0.513	Other
Ciddina	0.078	0.406	0.516	Other
Pizzuta	0.041	0.4	0.56	Other
Pizzutella	0.05	0.393	0.557	Other
Pasola	0.2	0.365	0.436	Other
Simone	0.016	0.354	0.631	Other
Nocella	0.044	0.343	0.613	Other
Geccollina	0.278	0.124	0.598	Other
Canua	0.253	0.057	0.69	Other
Limongella	0.219	0.189	0.592	Other
Oliva Nocciola	0.184	0.091	0.725	Other
Bianca	0.17	0.109	0.72	Other
Barone Di Monteprofico	0.155	0.018	0.827	Other
Spina	0.155	0.077	0.767	Other
Sanguinella	0.141	0.048	0.811	Other
Tonda Dolce	0.126	0.034	0.839	Other
Torremaggiorese	0.124	0.031	0.844	Other
Uccellina	0.11	0.212	0.678	Other
Pinzuta	0.103	0.19	0.707	Other
SX_71	0.099	0.032	0.869	Other
Moraiolo	0.069	0.021	0.91	Other
Fra Michele	0.067	0.022	0.912	Other
Grappolo	0.066	0.029	0.904	Other
Tunnella	0.064	0.017	0.918	Other
Nolca	0.061	0.047	0.891	Other
Arbequina	0.057	0.02	0.922	Other
Grappa	0.057	0.075	0.868	Other
Rumanella	0.055	0.022	0.923	Other
Tondina	0.052	0.018	0.93	Other
Trigna	0.051	0.156	0.793	Other
Rotondella	0.048	0.02	0.932	Other
Ornella	0.047	0.022	0.931	Other
Leucocarpa	0.047	0.082	0.871	Other
Rosciola	0.043	0.018	0.938	Other
Cannellino	0.036	0.016	0.948	Other
Lamanisi	0.036	0.036	0.928	Other
Fragolina	0.036	0.046	0.918	Other
SX_24	0.036	0.117	0.847	Other
Nociara	0.035	0.121	0.844	Other
Peranzana	0.032	0.044	0.924	Other
SX_6	0.03	0.036	0.935	Other
Rossola	0.029	0.02	0.951	Other
Dritta	0.027	0.059	0.915	Other
Ravece	0.027	0.102	0.871	Other
Cornola	0.021	0.053	0.926	Other
Cornulara	0.019	0.038	0.942	Other
SX_12	0.018	0.026	0.956	Other
Pizzutola	0.015	0.036	0.95	Other
Cellina di Nardò	0.014	0.022	0.964	Other
Saracina	0.013	0.017	0.969	Other

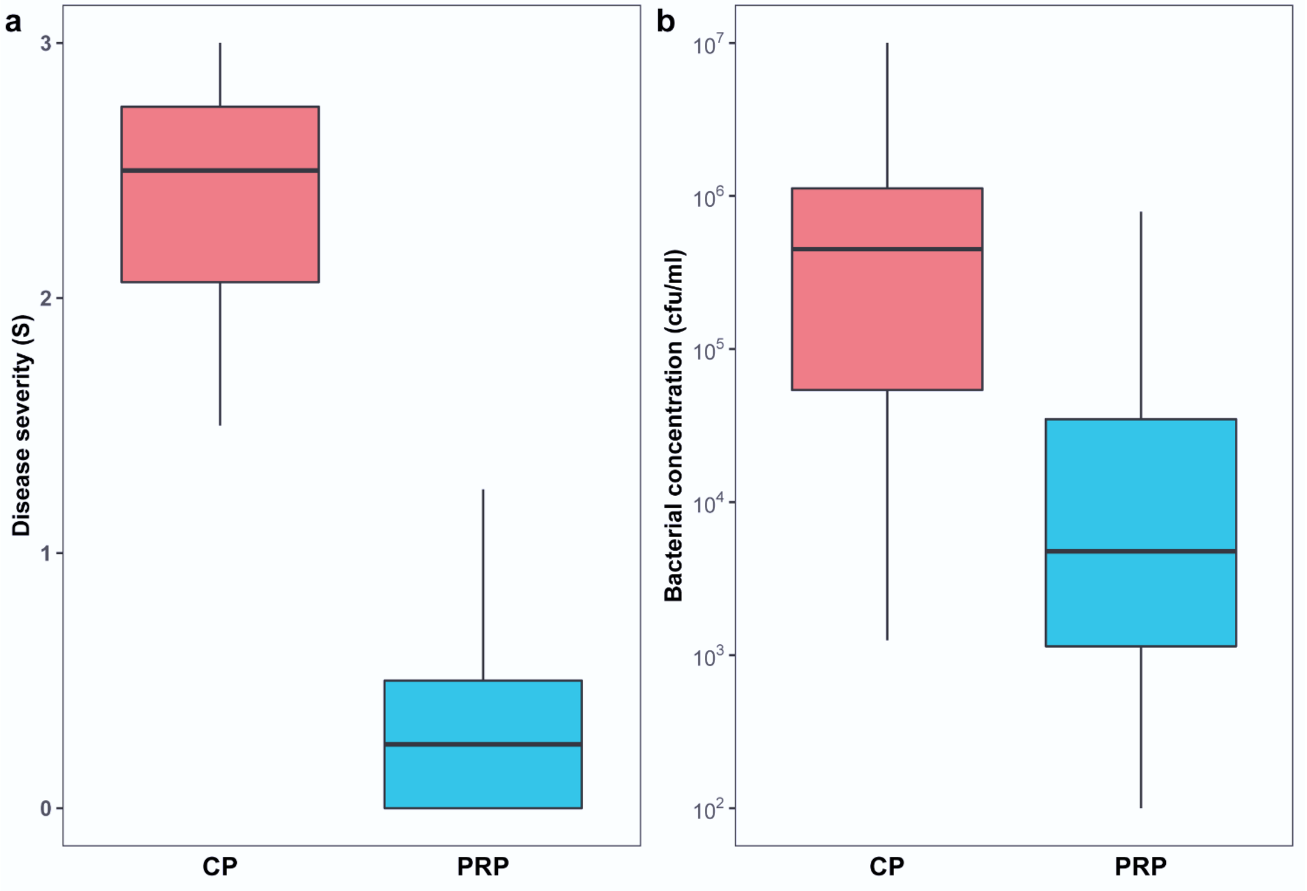

Supplementary Figure 1. Distribution of **(a)** disease severity scores (S) and **(b)** bacterial concentration (C) in thirty putatively resistant plants (PRPs) and respective control plants (CPs).

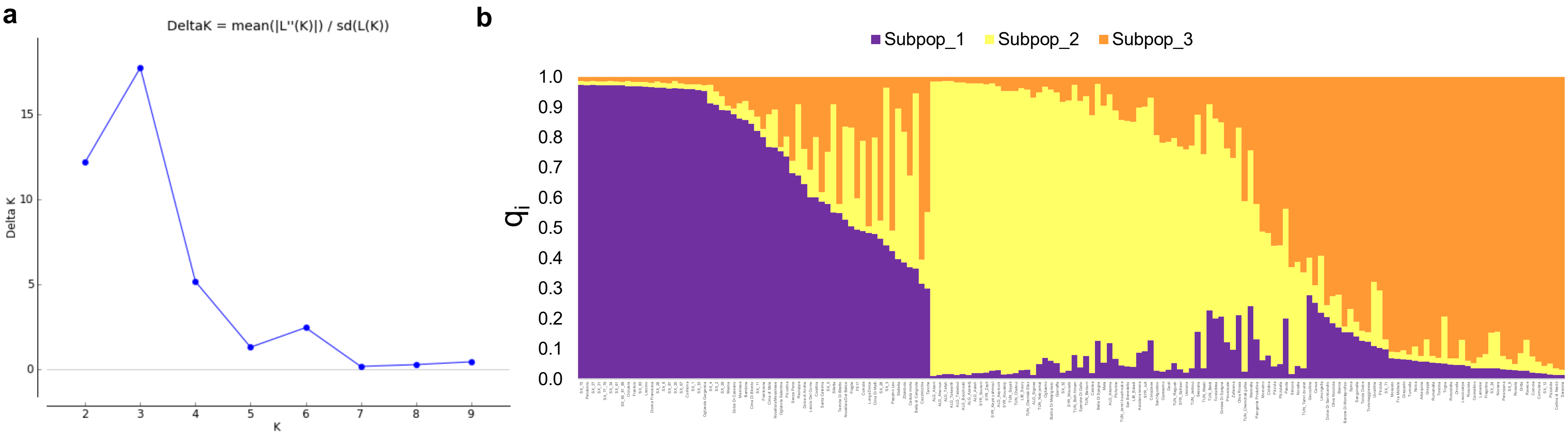

Supplementary Figure 2. Parametric study of genetic structure. **(a)** Distribution of the ΔK parameter. **(b)** Genetic structure for three subpopulations (K=3). Each individual is represented by a vertical line, which is partitioned into colored segments whose length is proportional to the estimated membership fraction (qi) in each subpopulation.

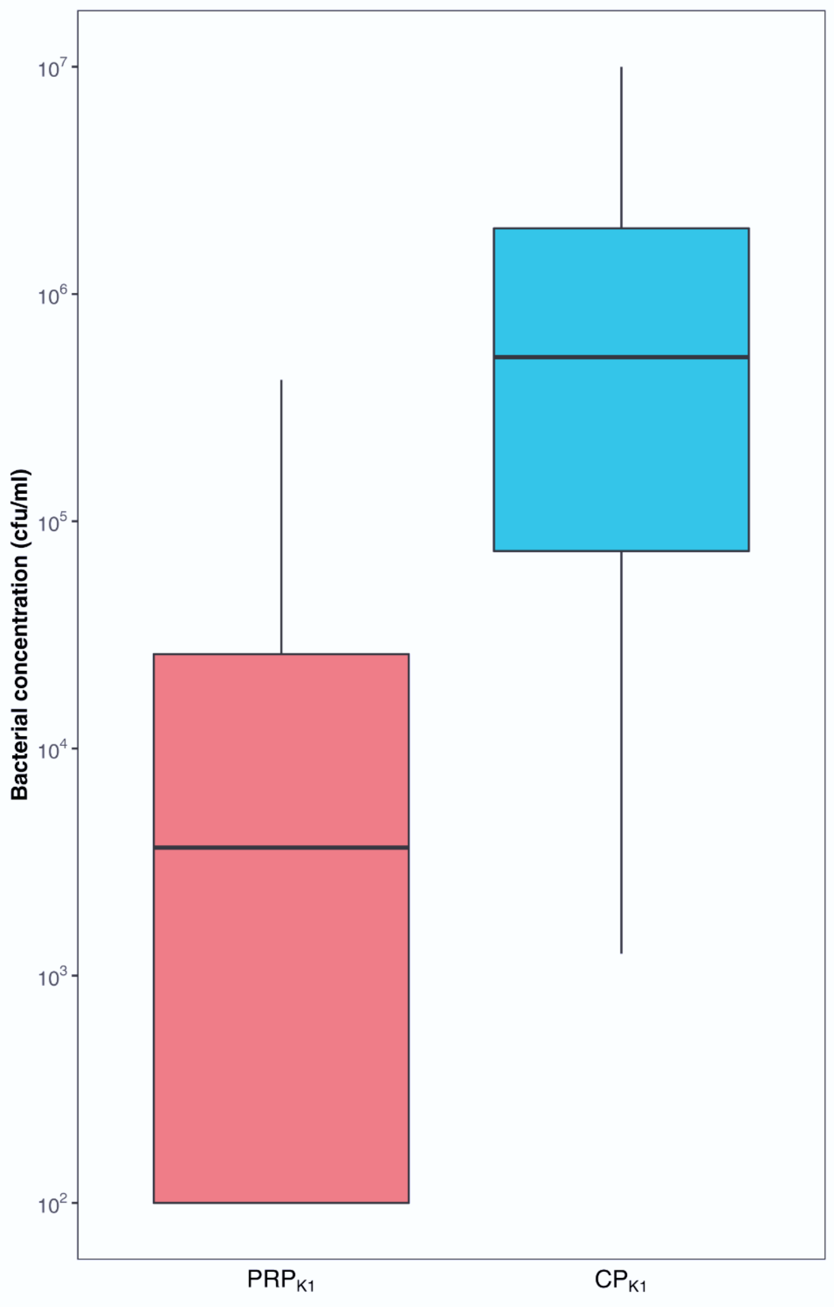

Supplementary Figure 3. Bacterial colonization levels in PRPs included in K1 with respect to control plants.
